# Comparative Outcomes of Lumbar Platelet-Rich Plasma Injection Versus Conservative Treatment for Chronic Discogenic and/or Radicular Pain: A Retrospective Cohort Study

**DOI:** 10.3390/biomedicines14051061

**Published:** 2026-05-07

**Authors:** Wen-Yuan Lee, Hao-Yuan Lee, Shu-Hua Ko, Po-Fan Chiu, Meng-Yen Li, Yu-Ling Huang, Chyi-Liang Chen

**Affiliations:** 1 Department of Neurosurgery, Wei Gong Memorial Hospital, Miaoli 35159, Taiwan; 005122@tool.caaumed.org.tw (W.-Y.L.); 030392@tool.caaumed.org.tw (P.-F.C.); 2 Department of Neurosurgery, China Medical University Hospital, China Medical University, Taichung 404327, Taiwan; 3 Department of Nursing, Jen-Teh Junior College of Medicine, Nursing and Management, Miaoli 35664, Taiwan; 4 Department of Pediatrics, Wei Gong Memorial Hospital, Miaoli 35159, Taiwan; 5 School of Medicine, College of Medicine, Fu Jen Catholic University, New Taipei 242062, Taiwan; 6 Division of Pediatric Infectious Diseases, China Medical University Children’s Hospital, China Medical University, Taichung 404327, Taiwan; 7 Molecular Infectious Disease Research Center, Chang Gung Memorial Hospital, Taoyuan 333423, Taiwan; 8 Department of Medical Quality, Wei Gong Memorial Hospital, Miaoli 35159, Taiwan; 045652@tool.caaumed.org.tw; 9 Spine Center, China Medical University Hospital, Taichung 404327, Taiwan; 10 Department of Medical Quality, Teaching and Research Section, Wei Gong Memorial Hospital, Miaoli 35159, Taiwan; 045441@tool.caaumed.org.tw (M.-Y.L.); 045870@tool.caaumed.org.tw (Y.-L.H.)

**Keywords:** discogenic pain, lumbar radicular pain, platelet-rich plasma, conservative treatment, numeric rating scale, Oswestry disability index

## Abstract

**Background/Objectives**: Few previous studies have evaluated both radiological changes and the clinical effectiveness of platelet-rich plasma (PRP) injections, a biomedical therapy, in patients with chronic discogenic and/or radicular low back pain. **Methods**: This retrospective comparative cohort study reviewed patients with chronic LBP (>3 months) refractory to first-line conservative therapy between July 2022 and August 2024. Patients who underwent lumbar transforaminal epidural or intradiscal PRP injections, according to clinical presentation and imaging findings, were assigned to the PRP group. (*n* = 312), while those continuing conservative treatment served as controls (*n* = 391). Patients receiving both treatments were excluded. Pain and functional outcomes were evaluated using the Numeric Rating Scale (NRS) and Oswestry Disability Index (ODI). Follow-up continued through February 2025. **Results**: Baseline demographic and clinical characteristics were comparable between groups (*p* > 0.05). At three months, the PRP group demonstrated significantly greater reductions in pain (NRS: 4.51 ± 0.79 from a baseline of 7.20 ± 0.89) and disability (ODI: 23.73 ± 3.96 from 37.21 ± 3.14), when compared with the conservative group (both *p* < 0.001). These improvements were sustained at six months (NRS: 2.86 ± 0.80 vs. 6.54 ± 1.99; ODI: 15.37 ± 3.99 vs. 33.70 ± 9.95; both *p* < 0.001). MRI changes were more frequent in the PRP group (73.09% vs. 9.28%, *p* < 0.001); however, these findings should be considered exploratory due to potential selection bias in imaging follow-up. **Conclusions**: PRP injection was associated with greater improvements in pain and function than conservative treatment. MRI improvements were more frequent in the PRP group, but these findings remain exploratory.

## 1. Introduction

In recent years, biomedical therapies such as platelet-rich plasma (PRP) treatment have garnered attention as potential regenerative solutions. PRP is an autologous preparation of platelets concentrated from venous blood, enriched with growth factors including platelet-derived growth factor (PDGF), vascular endothelial growth factor (VEGF), transforming growth factor-beta (TGF-β), and insulin-like growth factor-1 (IGF-1) [1,2,3,4,5,6,7]. These bioactive molecules regulate inflammation, promote angiogenesis, and enhance extracellular matrix synthesis, thereby supporting tissue repair [1,2,3,4,5,6,7]. Encouraging results have been reported in musculoskeletal conditions such as tendinopathy, osteoarthritis, and soft tissue injuries [8,9,10].

In the present study, we focus on discogenic pain and/or radicular pain associated with nerve root involvement, representing specific pathological subtypes of low back pain (LBP). LBP is one of the most prevalent conditions, affecting up to 80% of adults during their lifetime [11,12], and remains a leading cause of years lived with disability worldwide [13,14]. The burden is particularly pronounced in patients with structurally defined conditions such as discogenic and/or radicular pain, which are often associated with intervertebral disc degeneration, disc protrusion or herniation, and nerve root compression. These conditions are typically more persistent and functionally limiting than non-specific LBP, contributing substantially to healthcare utilization, reduced productivity, and long-term disability.

Conservative care remains the first-line treatment for patients with discogenic and radicular pain and typically includes patient education, activity modification, structured physical therapy, and pharmacological interventions such as nonsteroidal anti-inflammatory drugs (NSAIDs) or neuropathic agents [15,16]. Although these approaches aim to relieve pain and restore function, a substantial proportion of patients experience persistent or recurrent symptoms, leading to chronic low back pain [17]. In this study, chronic pain is defined as symptoms persisting for more than 12 weeks in patients with imaging or clinical findings consistent with discogenic and/or radicular pathology, while patients with only non-specific low back pain are excluded. However, it should be noted that prolonged medication use may be associated with adverse gastrointestinal, renal, or cardiovascular effects.

When conservative treatment fails, epidural steroid injections or surgery may be considered. However, corticosteroid injections generally provide only short-term relief, and repeated use may result in complications such as infection, tissue atrophy, or systemic side effects [18]. Surgical interventions, while effective in selected cases, are invasive, costly, and not without risk. These limitations highlight the need for alternative therapies that provide sustained benefit and target underlying degenerative processes.

In this context, PRP has been increasingly investigated as a biologically based treatment for spinal conditions with defined pathological mechanisms, particularly discogenic pain and lumbar radiculopathy. Randomized controlled trials have reported improvements in pain and functional outcomes following intradiscal PRP injections in patients with discogenic low back pain [19]. Epidural PRP injections have also been associated with clinical improvement in lumbar radiculopathy [20,21], while systematic reviews have suggested potential benefits in facet joint arthropathy [22]. Nevertheless, the overall evidence remains heterogeneous, with variability in study design, patient selection, injection techniques, and outcome measures leading to inconsistent findings across studies. Furthermore, emerging approaches such as combining PRP with mesenchymal stem cells or using advanced image-guided delivery techniques have been explored, but their added value remains to be fully established [23,24]. Collectively, while PRP shows promise, its clinical effectiveness in well-defined spinal pathologies such as discogenic and radicular pain has not been definitively established [3].

Importantly, most existing studies have compared PRP with corticosteroids, lidocaine, or placebo injections, or lacked appropriate non-injection control groups [25,26,27,28]. There is a paucity of evidence directly comparing PRP injections with structured conservative management in patients with discogenic and/or radicular pain, which represents the current standard first-line approach. Direct comparisons between PRP and structured conservative management without injection therapy are limited, and evidence regarding their relative effects on both clinical and radiological outcomes remains insufficient.

We hypothesized that PRP injections would be associated with greater improvements in pain, functional outcomes, and radiological parameters compared with structured conservative management in patients with chronic discogenic and/or radicular low back pain. Therefore, this study aimed to evaluate the comparative effectiveness and safety of PRP injections versus structured conservative management and assess associated radiological and clinical outcomes in this well-defined patient population.

## 2. Materials and Methods

### 2.1. Ethics Statement, Study Design, and Setting

This study was approved by the Institutional Review Board of China Medical University Hospital (IRB No. CMUH114-REC2-075) on 12 May 2025. Given the retrospective nature of the study, the requirement for written informed consent was waived. This retrospective cohort study was not prospectively registered, as it involved secondary analysis of existing clinical data. The study was conducted in accordance with the STROBE checklist (Table A1).

This retrospective cohort study was conducted at Wei Gong Memorial Hospital, Taiwan. Electronic health records from July 2022 to August 2024 were reviewed, and follow-up continued until February 2025. Patients were identified from hospital records and screened sequentially in accordance with predefined inclusion and exclusion criteria. The selection was based on all available cases that met the criteria, rather than on subjective sampling.

Treatment allocation was not randomized. Patients were assigned to the PRP group or the conservative treatment group based on shared decision-making between the treating physician and the patient as part of routine clinical practice. This decision was influenced by clinical factors, including symptom severity, imaging findings, functional impairment, patient preference, and financial considerations. No formal randomization or protocol-driven assignment was applied.

Given the retrospective nature of this study, no formal sample size calculation was performed. All patients who met the predefined inclusion and exclusion criteria during the study period were consecutively included to minimize selection bias.

### 2.2. Participants

**Figure 1:**
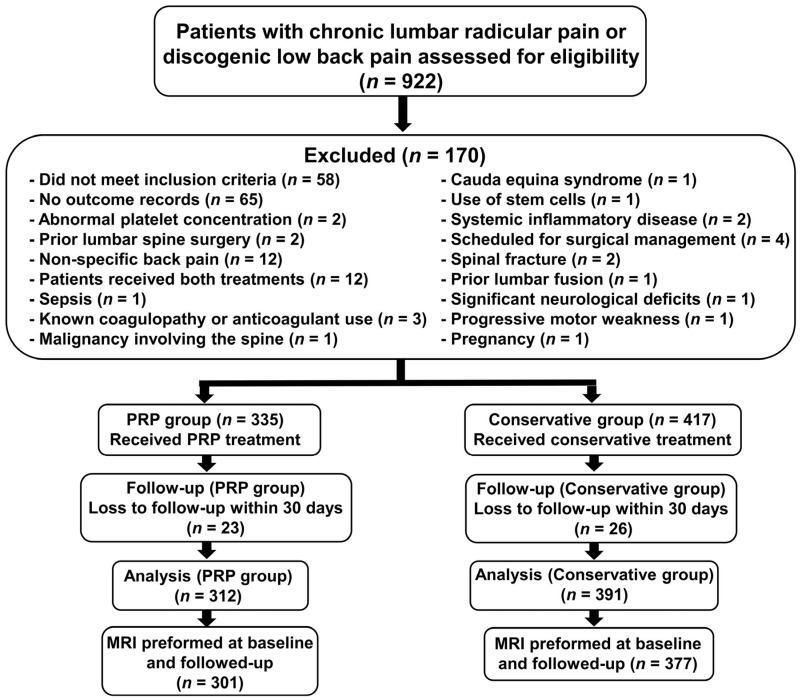
A study flow diagram. Patient flow diagram showing the number of patients assessed for eligibility, excluded with reasons, assigned to the PRP or conservative treatment cohorts, lost to follow-up, included in the final analysis, and undergoing baseline and follow-up MRI assessment.

Patients aged ≥18 years were eligible if they had chronic lumbar radicular pain or discogenic low back pain, confirmed by history, physical examination, and/or imaging (MRI or CT). Only patients with baseline and follow-up Numerical Rating Scale (NRS) and Oswestry Disability Index (ODI) scores were included (Figure 1). Patient-reported outcomes were assessed at baseline and at 3, and 6 months after treatment using ODI for disability, the NRS for pain intensity, the Patient Health Questionnaire-9 (PHQ-9) for depressive symptoms, and the Generalized Anxiety Disorder-7 (GAD-7) for anxiety.

Chronic discogenic and/or radicular pain in this study was defined as pain persisting for more than 12 weeks and restricted to cases with discogenic and/or radicular etiologies. Patients with non-specific or facet-mediated low back pain were excluded.

Patients were excluded if they had a history of prior lumbar spine surgery at the index level (including lumbar fusion or artificial disc replacement); systemic inflammatory disease; active infection (including spinal infection) or sepsis; malignancy involving the spine; pregnancy; known coagulopathy, anticoagulant use, or abnormal platelet concentration; or prior treatment with other biologic agents (e.g., stem cells or prolotherapy). Additional exclusion criteria included significant or progressive neurological compromise requiring urgent surgical evaluation (e.g., progressive motor weakness or cauda equina syndrome), as well as patients scheduled for surgical management during the study period based on standard clinical indications.

Patients who received both platelet-rich plasma (PRP) and conservative treatments were also excluded.

After applying these criteria, the final study cohort represented a non-surgical population with chronic discogenic and/or radicular low back pain, without major structural, infectious, neoplastic, or severe neurological conditions requiring immediate operative intervention.

As physicians did not record NRS or ODI scores for 65 patients, no outcome data were available in their medical records and these patients were therefore excluded from the final analysis. In addition, 49 patients were lost to follow-up within one month after baseline (23 in the PRP group and 26 in the conservative treatment group) and were also excluded.

### 2.3. PRP Group

Patients who received PRP treatment without any conservative therapy were classified into the PRP group. PRP preparation followed a standardized double-spin centrifugation protocol. Between 30 and 60 mL of autologous venous blood was collected into anticoagulated tubes, centrifuged to separate plasma fractions, and further processed to yield leukocyte-poor PRP with a platelet concentration 3–5 times baseline. Approximately 2–4 mL of PRP was administered per spinal level using either an ultrasound-guided lumbar transforaminal epidural approach or a fluoroscopy-assisted intradiscal approach, depending on the clinical presentation and target tissue [29,30]. The treated spinal segment was selected by correlating the patient’s primary pain pattern with physical examination findings and Magnetic resonance imaging (MRI) abnormalities. The level showing the strongest clinical–radiologic concordance, such as pain provocation on examination and corresponding degenerative changes on MRI, was identified as the primary pain generator and targeted for PRP injection. Needle positioning was confirmed using anteroposterior and lateral views; contrast injection was performed in selected cases to verify accurate spread. Local infiltration with 1–2% lidocaine was used for patient comfort. PRP was not externally activated before injection, and activation was assumed to occur in vivo after administration. Following injection, patients were monitored for 30–60 min before discharge. Repeat injections were permitted if patients experienced only partial but clinically meaningful improvement after the initial injection, persistent or recurrent pain at the same symptomatic level, or a return of symptoms following an initial response; however, most analyses were based on a single injection episode. PRP injections delivered using an ultrasound-guided lumbar transforaminal epidural approach have been validated for safety and accuracy in previous studies [29,30].

### 2.4. Conservative Group

The conservative treatment group received guideline-concordant management including patient education, activity modification, and a structured home- or clinic-based exercise program focused on core stabilization and lumbar flexibility. Pharmacological treatment included nonsteroidal anti-inflammatory drugs (NSAIDs) and/or neuropathic pain medications when clinically indicated. Physical therapy, when prescribed, typically consisted of supervised sessions 1–2 times per week, combined with daily home exercises. Treatment duration was individualized and generally continued for 4–6 months, with regular outpatient follow-up to monitor symptoms and functional status. Adherence to exercise and medication use was assessed via patient self-report at follow-up visits.

In our cohort, all patients across the included disease categories received similar pharmacological management, primarily consisting of oral analgesics, including NSAIDs, acetaminophen, and neuropathic agents. None of the patients in this group received injection-based therapies during the observation period. Conservative treatment followed guideline-concordant multimodal care, reflecting current evidence-based recommendations [15,16].

### 2.5. Baseline Comparison of the PRP Group and Conservative Group

Baseline characteristics, including age, sex, family income quartile, prior pain duration (months), pain type (discogenic, radicular, or mixed), MRI findings (spinal stenosis, disc degeneration, disc protrusion/herniation, or no MRI available), baseline NRS, baseline ODI, baseline GAD-7, and baseline PHQ-9 were compared between the PRP and conservative treatment group to assess potential confounders.

### 2.6. Definitions

Discogenic pain was defined based on clinical history, physical examination, and imaging findings (MRI and/or CT) compatible with discogenic and/or radicular pathology, including chronic low back pain arising from a degenerated intervertebral disc. Typical imaging features included findings such as a high-intensity zone (HIZ) on T2-weighted images in the posterior annulus. Provocative discography was not routinely performed in this cohort and was therefore not required for diagnosis [31]. Radicular pain is pain caused by mechanical compression and/or inflammation of a spinal nerve root, often presenting as sharp, shooting, or electric-like pain radiating along a dermatome, sometimes accompanied by sensory or motor changes [32].

### 2.7. Outcomes

The primary outcome was back pain intensity, measured by NRS (0 = no pain, 10 = worst imaginable pain). The secondary outcome was functional disability, assessed using the ODI (0–100, higher scores indicate greater disability). Both were measured at baseline, 3 months, and 6 months. Adverse events (e.g., infection, neurological injury, hospitalization) and psychological conditions, such as anxiety or depression were recorded using patient charts. GAD-7 and PHQ-9 were prespecified exploratory variables rather than primary or secondary efficacy endpoints.

MRI was performed in patients in both the PRP and conservative treatment groups. Examinations were obtained at baseline and ≥45 days after PRP injection using a standardized lumbar spine protocol. The images were descriptively reviewed to evaluate changes in intervertebral disc signal characteristics and neural canal morphology. All imaging evaluations were independently reviewed by two neurosurgeons.

MRI findings were classified using categorical criteria (improved/unchanged/worsened) based on standardized radiological definitions of disc morphology and signal changes. MRI improvement was defined as changes in disc morphology, including reduced disc protrusion, decreased annular fissure, or improved disc hydration on T2-weighted imaging, as assessed by a follow-up MRI. All MRI images were independently reviewed by two experienced physicians who were blinded to clinical outcomes and treatment allocation. Inter-rater agreement was evaluated using Cohen’s kappa coefficient.

Follow-up MRI was not routinely performed in all patients but was conducted in a subset based on clinical indications, including persistent symptoms or the need for further evaluation. Therefore, imaging outcomes were analyzed descriptively and interpreted with caution.

### 2.8. Statistical Analysis

Data were analyzed using SPSS version 26.0 (IBM Corp., Armonk, NY, USA). Continuous variables are presented as mean ± standard deviation and were compared using independent *t*-tests. Categorical variables are summarized as frequencies (%) and were compared using chi-square tests. Statistical significance was set at *p* < 0.05 (two-sided). Repeated-measures analysis of variance (ANOVA) was performed to assess the effects of group, time, and the group × time interaction. In addition, 95% confidence intervals (CIs) are reported to improve interpretability. A linear mixed-effects model was used to evaluate changes in outcomes (NRS and ODI) over time between the PRP and conservative treatment groups. The model included a random intercept for each patient (clustering unit), with fixed effects for treatment group, time, and prespecified covariates, as well as the treatment × time interaction. An unstructured covariance matrix was specified to model within-subject correlations, and parameters were estimated using restricted maximum likelihood (REML).

## 3. Results

### 3.1. Patient Characteristics

A total of 703 patients met the eligibility criteria, of whom 312 received PRP and 391 underwent conservative treatment (Figure 1). Table 1 summarizes the baseline characteristics. The mean age was 64.6 ± 10.2 years in the PRP group and 64.4 ± 9.0 years in the conservative group, with no significant difference (*p* = 0.807). The sex distribution was balanced between groups (PRP: 157 males and 155 females; conservative: 196 males and 195 females; *p* = 0.960). Baseline NRS (7.20 ± 0.89 vs. 7.17 ± 0.78, *p* = 0.599) and ODI (37.21 ± 3.14 vs. 37.27 ± 2.54, *p* = 0.781) scores were comparable, indicating well-matched groups.

Additional clinically relevant variables, including symptom duration, prior treatments (e.g., lumbar surgery and PRP therapy), and socioeconomic status (categorized into quartiles), prior pain duration (months), pain type (discogenic, radicular, or mixed), MRI findings (spinal stenosis, disc degeneration, disc protrusion/herniation, or no MRI available), baseline NRS, baseline ODI, baseline GAD-7, and baseline PHQ-9 are included in Table 1. No significant differences were observed between the PRP and conservative groups for these variables, supporting comparability at baseline.

Overall, there were no significant differences in baseline characteristics, including age, sex, diagnoses, symptom duration, prior treatments, and socioeconomic status, between the two groups, suggesting that these factors were unlikely to act as major confounders in this study.

Among the 312 patients in the PRP treatment group, the predominant symptom patterns were as follows: 94 (30.13%) had pure discogenic pain, 61 (19.55%) had pure radicular pain, and 157 (50.32%) presented with mixed pain. PRP injections were administered at the L4–S1 levels in 188 patients (60.26%), at the L3–L4 levels in 63 patients (20.19%), and at the L1–L3 levels in 61 patients (19.55%).

Among the 301 patients who underwent MRI evaluation, imaging-defined pathologies included lumbar disc protrusion or herniation in 124 patients (41.20%), lumbosacral disc degeneration in 82 patients (27.24%), and spinal stenosis in 95 patients (31.56%). Among the 95 patients with spinal stenosis, 59 (62.11%) had moderate stenosis (aggregation of the cauda equina, making individual nerve roots difficult to distinguish), 33 (34.74%) had mild stenosis (minor crowding with preservation of separation among cauda equina roots), and 3 (3.16%) had severe stenosis (complete effacement of cerebrospinal fluid, with the cauda equina appearing as a single bundle) [33].

Among the 391 patients in the conservative treatment group, 273 (69.82%) underwent supervised physical therapy, 294 (75.19%) were prescribed home exercise programs, 264 (67.52%) received nonsteroidal anti-inflammatory drugs (NSAIDs), 255 (65.22%) received acetaminophen, and 129 (33.00%) received neuropathic medications. Over the 6-month study period, the median duration of conservative treatment was 171 days, and patients had 2–6 follow-up visits (median, 3 visits).

### 3.2. Pain Outcomes

**Figure 2:**
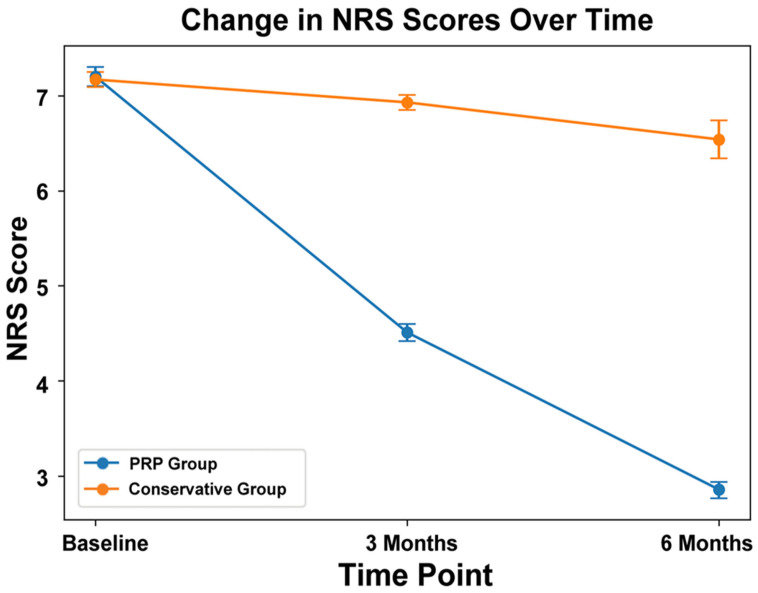
Comparison of mean Numeric Rating Scale (NRS) scores between the platelet-rich plasma (PRP) group and the conservative treatment group at baseline, 3 months, and 6 months, with error bars representing the 95% confidence intervals.

At 3 months, NRS scores decreased significantly more in the PRP group than in the conservative treatment group (down to 4.51 ± 0.79 vs. 6.93 ± 0.78, 95% CI: 4.42–4.60 vs. 6.85–7.01, *p* < 0.001). At 6 months, the PRP group demonstrated sustained improvement, with a mean NRS score of 2.86 ± 0.80 (95% CI: 2.77–2.94) compared with 6.54 ± 1.99 (95% CI: 6.34–6.74) in the conservative group (*p* < 0.001) (Figure 2). The absolute reduction in NRS score from baseline was 4.35 points in the PRP group, compared with 0.63 points in the conservative group.

### 3.3. Functional Outcomes

**Figure 3:**
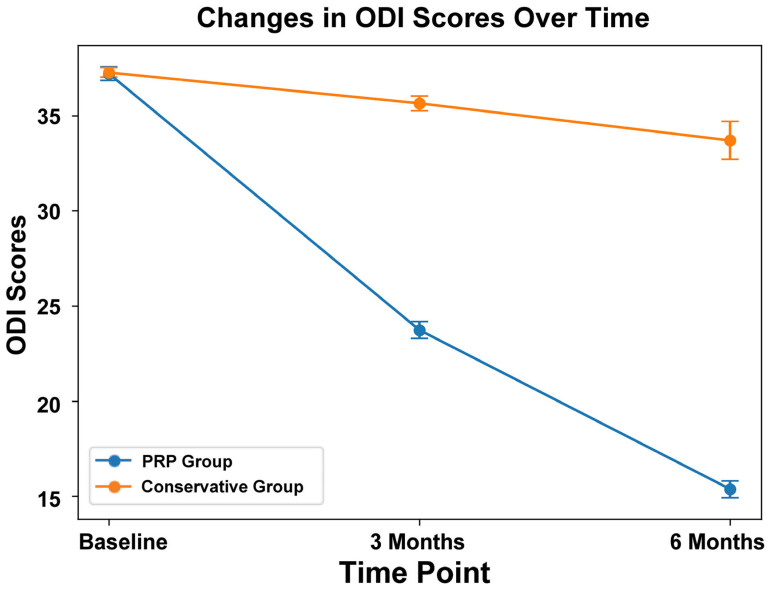
Comparison of mean Oswestry Disability Index (ODI) scores between the platelet-rich plasma (PRP) group and the conservative treatment group at baseline, 3 months, and 6 months, with error bars representing the 95% confidence intervals.

At 3 months, the mean ODI score was significantly lower in the PRP group than in the conservative group (23.73 ± 3.96 vs. 35.65 ± 3.91, 95%CI: 23.29–24.18 vs. 35.26–36.04, *p* < 0.001). Further improvement was observed at 6 months in the PRP group, with a mean ODI of 15.37 ± 3.99 (95%CI: 14.92–15.81), whereas the conservative group showed minimal change (33.70 ± 9.95, 95%CI: 32.71–34.69, *p* < 0.001) (Figure 3). Overall, this corresponded to a 21.8-point reduction in disability in the PRP group compared with a 3.6-point reduction in the conservative treatment group.

### 3.4. Clinical Relevance of the Results

At three months, the PRP group demonstrated significantly greater reductions in pain (NRS: 4.51 ± 0.79 from a baseline of 7.20 ± 0.89) and disability (ODI: 23.73 ± 3.96 from 37.21 ± 3.14), when compared with the conservative group (both *p* < 0.001). These changes exceeded the established minimal clinically important differences (MCID) for both NRS and ODI. These improvements were sustained at six months (NRS: 2.86 ± 0.80 vs. 6.54 ± 1.99; ODI: 15.37 ± 3.99 vs. 33.70 ± 9.95; both *p* < 0.001), and remained above MCID thresholds, indicating clinically meaningful benefit in addition to statistical significance. Minimal clinically important difference (MCID) was defined as a reduction of ≥2 points in the Numerical Rating Scale (NRS) for pain and ≥10 points in the Oswestry Disability Index (ODI), based on previously established thresholds in patients with chronic low back pain [34].

### 3.5. Repeated-Measures Analysis of Variance (ANOVA)

A repeated-measures ANOVA was performed to evaluate the effects of treatment method (PRP vs. conservative), time (baseline, 3 months, and 6 months), and their interaction on Numeric Rating Scale (NRS) scores. The means, standard deviations (SDs), and 95% confidence intervals (CIs) for NRS scores are presented in Table 2. Mauchly’s test indicated that the assumption of sphericity was violated (χ^2^ (2) = 257.578, *p* < 0.001); therefore, the degrees of freedom were corrected using the Huynh–Feldt estimate (ε = 0.767). The repeated-measures ANOVA demonstrated a statistically significant effect of treatment over time, including a significant group × time interaction (F (1.534, 1075.384) = 197.883, *p* < 0.001).

A repeated-measures ANOVA was also conducted to assess the effects of treatment method, time, and their interaction on Oswestry Disability Index (ODI) scores. The means, SDs, and 95% CIs for ODI scores are presented in Table 2. Mauchly’s test indicated a violation of the sphericity assumption (χ^2^ (2) = 257.578, *p* < 0.001); therefore, the Greenhouse–Geisser correction was applied (ε = 0.713). The analysis showed a statistically significant effect of treatment over time, including a significant group × time interaction (F (1.421, 996.213) = 1172.661, *p* < 0.001).

### 3.6. Mixed-Effects Model

Outcomes (NRS and ODI) and 13 covariates were analyzed using a mixed-effects model (Table 3). Only treatment (PRP vs. conservative care) was significantly associated with both outcomes (NRS and ODI; both *p* < 0.001). Age, sex, family income quartile, prior pain duration (months), pain type (discogenic, radicular, or mixed), MRI findings (spinal stenosis, disc degeneration, disc protrusion/herniation, or no MRI available), PRP injection site (L4–S1, L3–L4, L1–L3, or conservative treatment), treatment modality (transforaminal epidural PRP, intradiscal PRP, or conservative care), baseline NRS, baseline ODI, baseline GAD-7, and baseline PHQ-9 were not significantly associated with the outcomes (all *p* > 0.05).

In the PRP group, patients received either transforaminal epidural PRP injections or intradiscal PRP injections based on clinical presentation and imaging findings. A total of 81 patients underwent transforaminal epidural PRP injections, whereas 231 patients received intradiscal PRP injections. Each patient was treated at the affected spinal level (s) (L1–S1) according to symptom distribution and imaging correlation. Repeat injections were performed in 15 patients based on clinical response, with a maximum of two injections during the study period. Subgroup analyses according to injection technique (transforaminal vs. intradiscal) were conducted for clinical outcomes (NRS and ODI). No significant differences were observed between the two approaches in the mixed-effects model (Table 3) or in independent *t*-tests (Table A2).

### 3.7. Adverse Events

No major adverse events such as infection, neurological deficit, or hospitalization occurred. Minor transient post-injection soreness events were reported in 16 PRP patients (5.1%), all of which resolved within 48–72 h without additional treatment. According to our retrospective records, no patients in the PRP group were newly diagnosed with psychiatric conditions, such as anxiety or depression, by psychiatrists during the study period following PRP treatment.

### 3.8. Changes in Psychological Scale Scores

GAD-7 and PHQ-9 were prespecified experimental results rather than primary or secondary efficacy endpoints. The GAD-7 was assessed at baseline and at 6 months. In the PRP group, GAD-7 scores decreased significantly from 6.20 ± 1.42 (95% CI: 6.04–6.36) at baseline to 3.02 ± 0.77 (95% CI: 2.93–3.10) at 6 months (*p* < 0.001). In contrast, the conservative treatment group showed minimal change, with scores of 6.26 ± 1.93 (95% CI: 6.12–6.40) at baseline and 6.04 ± 1.23 (95% CI: 5.92–6.16) at 6 months. At 6 months, GAD-7 scores were significantly higher in the conservative group than in the PRP group (*p* < 0.001).

Similarly, the PHQ-9 was assessed at baseline and at 6 months. In the PRP group, PHQ-9 scores decreased significantly from 6.48 ± 1.56 (95% CI: 6.34–6.62) at baseline to 4.69 ± 1.07 (95% CI: 4.57–4.81) at 6 months (*p* < 0.001). In contrast, the conservative treatment group demonstrated minimal change, with scores of 6.47 ± 1.26 (95% CI: 6.34–6.59) at baseline and 6.54 ± 1.26 (95% CI: 6.42–6.67) at 6 months. At 6 months, PHQ-9 scores were significantly higher in the conservative group than in the PRP group (*p* < 0.001).

### 3.9. Magnetic Resonance Imaging Findings

**Figure 4:**
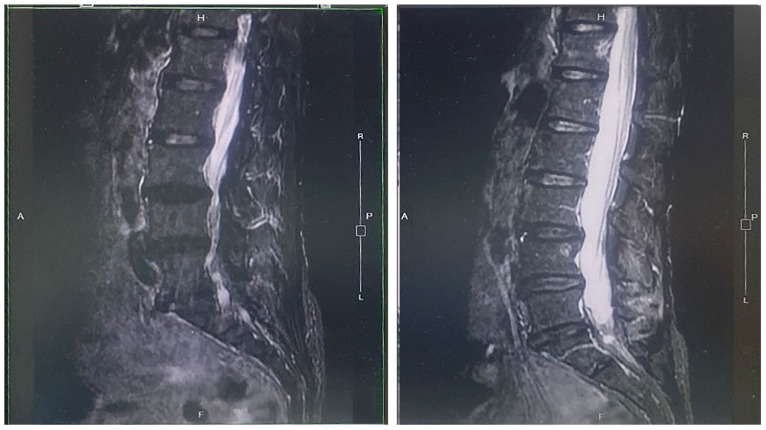
Magnetic resonance imaging findings before and after platelet-rich plasma (PRP) treatment. At baseline, the intervertebral disc exhibited degenerative changes, including reduced hydration and decreased T2-weighted disc signal intensity (**left**). At 45 days after the PRP injection, the neural canal appeared to have widened, and the intervertebral disc demonstrated increased signal intensity relative to baseline (**right**). This image represents a single illustrative case and should be interpreted as a representative example rather than evidence of uniform structural improvement across the entire cohort.

The MRI shown in Figure 4 was obtained from a patient in the PRP group. At baseline, the intervertebral disc exhibited degenerative features, including reduced hydration and decreased T2-weighted disc signal intensity. At 45 days after PRP injection, MRI demonstrated improvements, including widening of the neural canal and increased intervertebral disc signal intensity compared with baseline. Among patients who underwent follow-up MRI (301 in the PRP group and 377 in the conservative treatment group), MRI improvements were observed in a greater proportion of patients in the PRP group (220/301, 73.09%) than in the conservative treatment group (35/377, 9.28%) (*p* < 0.001). Interobserver agreement for MRI assessment was excellent, with a Cohen’s kappa of 1.000.

In the subgroup of patients who underwent follow-up MRI, a proportion demonstrated imaging changes consistent with the predefined criteria for MRI improvement. However, given that imaging was available only for a subset of patients and not performed systematically, these findings should be interpreted as exploratory rather than definitive.

## 4. Discussion

This study evaluated, in a real-world cohort of patients with discogenic and/or radicular pain, the association between PRP injections and clinical and radiological outcomes over a 6-month follow-up period. PRP injections were associated with improvements in pain and function compared with conservative treatment. However, given the retrospective design, these findings should be interpreted with caution and do not establish causality.

### 4.1. Principal Findings

The primary finding of this study is that patients treated with PRP experienced greater reductions in pain and disability scores over 6 months compared with those receiving structured conservative care. MRI changes were also observed more frequently in the PRP group; however, these imaging findings were based on a clinically selected subset of patients and are therefore considered exploratory.

### 4.2. Comparison with the Prior Literature

The demographics of our cohort align with global epidemiology of chronic low back pain, which predominantly affects middle-aged and older adults with near-equal sex distribution [11,22].

Baseline comparability between groups on key demographic and diagnostic variables provides some support for internal validity; however, the absence of other prognostic factors and the retrospective nature of the study limits the extent to which confounding bias can be excluded.

While the seminal randomized controlled trial by Tuakli-Wosornu et al. reported a statistically significant improvement in best pain (NRS) at 8 weeks in the PRP group compared with the contrast control group, the benefit was limited to this single outcome at an early time point [27]. Later randomized trials, such as that by Schepers et al., found no significant difference between PRP and control in average pain, worst pain, or disability measures [35], highlighting the inconsistency of prior findings.

Systematic reviews and meta-analyses also reflect this uncertainty, noting that although intradiscal PRP may relieve pain, the overall quality of evidence is low, and many trials show only modest or transient effects [36]. Importantly, prior studies differ substantially in PRP preparation, injection technique, and patient selection, which likely contributes to inconsistent findings across trials.

Although some retrospective studies using higher-concentration PRP have reported more durable improvements in pain and function [37], these findings are not consistent across the literature and should be interpreted cautiously.

### 4.3. Interpretation of Current Findings and Discrepancy with Prior Studies

The magnitude of pain reduction observed in our PRP group (approximately 4-point decrease in NRS at 6 months) is clinically notable; however, this effect size should be interpreted in the context of potential selection bias, residual confounding, placebo effects, and expectation bias inherent to retrospective designs.

Differences between our findings and prior randomized trials may be explained by several factors. First, our study reflects a real-world population with broader inclusion of discogenic and radicular pain phenotypes. Second, PRP preparation and delivery were not fully standardized, potentially which may have influenced biological activity. Third, comparator treatment in our study reflects heterogeneous but structured conservative care rather than strict protocolized controls used in randomized trials. Finally, non-randomized treatment allocation may have resulted in the selection of patients more likely to respond to biologic interventions.

Together, these factors suggest that while the observed outcomes are encouraging, the level of evidence remains limited and should be interpreted cautiously in light of existing randomized and systematic evidence.

Previous studies, including those by Tuakli-Wosornu et al. and Akeda et al. [27,38], have also demonstrated sustained improvements in pain and function after intradiscal PRP injections. Nevertheless, the variability in study designs and outcomes across the literature underscores the need for cautious interpretation of apparent treatment effects.

### 4.4. Surgical Context and Clinical Positioning

At our institution, surgical intervention is offered only when standard clinical indications are met—most commonly imaging-confirmed nerve-root compression that correlates with symptoms, progressive or functionally limiting motor deficit, persistent and intolerable pain despite structured conservative management, or urgent conditions such as cauda equina syndrome [39,40].

Randomized trials have shown that early surgery provides faster symptom relief, although long-term outcomes are often comparable to prolonged conservative management, with a substantial proportion of patients crossing over to surgery due to persistent pain [41]. Patient preference remains a major determinant of treatment selection [40].

To avoid crossover bias, patients scheduled for surgery during the study period were excluded. Therefore, the study population represents a subset of patients with severe pain who did not undergo immediate surgical intervention, rather than the full spectrum of surgically eligible patients.

While PRP may represent a potential intermediate option between conservative therapy and surgery, this hypothesis remains speculative and cannot be confirmed in the present study. Any suggestion that PRP reduces or delays surgical need should be considered exploratory.

### 4.5. Mechanistic Rationale

The regenerative potential of PRP lies in its concentration of growth factors that modulate inflammatory pathways and stimulate repair; for example, PDGF promotes fibroblast proliferation, VEGF supports neovascularization, and TGF-β enhances extracellular matrix deposition [1,2,3,4,5,6,7,8]. Preclinical studies have demonstrated that PRP stimulates proliferation of nucleus pulposus cells, upregulates type II collagen and aggrecan synthesis, and suppresses proinflammatory cytokines such as TNF-α and IL-1β [1,2,3,4,5,6,7,8].

### 4.6. Clinical Implications

These findings have potential clinical relevance. PRP may be considered in patients with persistent discogenic and/or radicular pain who are unresponsive to conservative measures but who are unwilling or unfit for surgery.

Patient selection appears critical, with prior evidence suggesting better outcomes in individuals with mild-to-moderate degeneration and without severe structural instability.

Shared decision-making should emphasize PRP’s autologous nature, minimal systemic risks, and its uncertain but potentially regenerative benefits.

### 4.7. Strengths and Limitations

This study has several strengths, including its real-world design, relatively large cohort, and longitudinal assessment of both pain and functional outcomes.

Several limitations must be acknowledged. First, as a retrospective cohort study, the possibility of selection bias, such as the exclusion of patients with missing data, cannot be ruled out. Although baseline characteristics were comparable, unmeasured confounders remain a major limitation.

Second, our follow-up period was limited to 6 months; longer-term outcomes beyond one year are essential to evaluate durability. Third, variations in PRP preparation protocols across institutions complicate cross-study comparisons [42]; in particular, heterogeneity in PRP preparation and injection technique (transforaminal epidural versus intradiscal) may have influenced outcomes. Fourth, a follow-up MRI was performed only in a subset of patients based on clinical indication, introducing selection bias; therefore, the radiological findings should be considered exploratory and hypothesis-generating rather than confirmatory evidence of tissue regeneration. Fifth, a total of 65 patients with missing outcome data (NRS/ODI) and 49 patients lost to follow-up within one month were excluded from the final analysis, which may introduce selection bias and limit the generalizability of the findings. Sixth, changes in anxiety and depression scores should be interpreted cautiously, as they may be influenced by pain improvement, placebo or expectation effects, and potential unmeasured confounding between groups.

Additionally, key prognostic variables such as smoking status, psychological comorbidities, pain catastrophizing, and socioeconomic factors were not available, introducing further potential confounding.

As this was a retrospective non-randomized study, placebo effects and expectation bias cannot be excluded. Future randomized controlled trials incorporating sham procedures and standardized PRP protocols are required to establish causal efficacy.

## 5. Conclusions

To the best of our knowledge, this is among the first real-world cohort studies to directly compare clinical outcomes and radiological changes between PRP and structured non-injection conservative therapy in a real-world cohort of patients with discogenic and/or radicular pain. PRP injections were associated with greater improvements in pain and functional outcomes in this cohort, when compared with conservative management. Although radiological changes were observed more frequently in the PRP group, these findings should be interpreted cautiously due to selection bias in imaging follow-up. Furthermore, given the retrospective and non-randomized study design, the presented findings should be interpreted as associative rather than causal, and as hypothesis-generating rather than evidence of treatment efficacy. These benefits appeared to be sustained for at least six months and were accompanied by minimal adverse effects. Further high-quality prospective randomized controlled trials with standardized protocols and longer follow-up are required to confirm these observations, clarify patient selection, and determine true clinical effectiveness.

## Figures and Tables

**Table 1:** Baseline background characteristics by treatment.

Characteristics		PRP Group (*N* = 312) *N* (%)	Conservative Group (*N* = 391) *N* (%)	*p* Value
Age, mean (SD) (yr)		64.67 (10.32)	64.60 (10.72)	0.937
Female sex		157 (50.32)	196 (50.13)	0.960
Prior pain duration, mean (SD) (months)		7.56 (6.25)	7.59 (6.34)	0.941
Pure discogenic pain		94 (30.13)	118 (30.18)	0.497
Pure radicular pain		61 (19.55)	77 (19.69)	0.962
Both discogenic and radicular pain		157 (50.32%)	197 (50.38)	0.987
Prior lumbar spine surgery		0 (0)	0 (0)	1.000
Prior PRP treatment before the study period		0 (0)	0 (0)	1.000
Family income, quartile				
<Q1		77 (24.68)	98 (25.06)	0.930
Q1–Q2		78 (25.00)	98 (25.06)	1.000
Q2–Q3		79 (25.32)	97 (24.81)	0.930
>Q3		78 (25.00)	98 (25.06)	1.000
NRS, mean (SD)		7.20 (0.89)	7.17 (0.78)	0.564
NRS, 95% CI		7.10–7.30	7.09–7.25	0.564
ODI score, mean (SD)		37.21 (3.14)	37.27 (2.54)	0.781
ODI, 95% CI		36.86–37.56	37.02–37.52	0.781
GAD-7, mean (SD)		6.20 (1.42)	6.26 (1.93)	0.596
GAD-7, 95% CI		6.04–6.36	6.12–6.40	0.596
PHQ-9, mean (SD)		6.48 ± 1.56	6.47 ± 1.26	0.894
PHQ-9, 95% CI		6.34–6.62	6.34–6.59	0.894
MRI-defined pathology, stratified by sex (male [M] and female [F])				
Spinal stenosis	M	45 (14.42)	56 (14.32)	0.970
	F	50 (16.02)	63 (16.11)	0.975
Degeneration of the lumbosacral disc	M	52 (16.67)	65 (16.62)	0.988
	F	30 (9.62)	38 (9.72)	0.963
Lumbar disc protrusion or herniation	M	53 (16.99)	66 (16.88)	0.970
	F	71 (22.76)	89 (22.76)	0.999
No MRI performed	M	5 (1.60)	8 (2.05)	0.782
	F	6 (1.92)	6 (1.53)	0.774

Abbreviations: PRP, platelet-rich plasma; N, total number; SD, standard deviation; 95% CI, 95% confidence interval; M, male; F, female.

**Table 2:** Descriptive statistics of Numeric Rating Scale (NRS) and Oswestry Disability Index (ODI) scores in the PRP and conservative treatment groups over time.

Characteristics	PRP Group			Conservative Group		
	Mean	SD	95% CI	Mean	SD	95% CI
NRS (at 0 month)	7.20	0.89	7.10–7.30	7.17	0.78	7.09–7.25
NRS (at 3 month)	4.51	0.79	4.42–4.60	6.93	0.78	6.85–7.01
NRS (at 6 month)	2.86	0.80	2.77–2.94	6.54	1.99	6.34–6.74
ODI (at 0 month)	37.21	3.14	36.86–37.56	37.27	2.54	37.02–37.52
ODI (at 3 month)	23.73	3.96	23.29–24.18	35.65	3.91	35.26–36.04
ODI (at 6 month)	15.37	3.99	14.92–15.81	33.70	9.95	32.71–34.69

Abbreviations: PRP, platelet-rich plasma; SD, standard deviation; 95% CI, 95% confidence interval; NRS, Numeric Rating Scale; ODI, Oswestry Disability Index.

**Table 3:** Estimates of the associations between outcomes (NRS and ODI) and 13 covariates from a mixed-effects model.

Variables	NRS			ODI		
	Estimate	*p* Value	95% CI	Estimate	*p* Value	95% CI
Treatment (PRP vs. Conservative)	−2.508	<0.001	−2.878–−2.138	−12.197	<0.001	−14.043–−10.350
Age	−0.000	0.896	−0.006–0.005	−0.003	0.824	−0.029–0.023
Sex	0.060	0.287	−0.051–0.170	−0.347	0.219	−0.206–0.900
Family income, quartile	−0.026	0.308	−0.076–0.024	−0.120	0.342	−0.369–0.128
Prior pain duration (month)	0.002	0.714	−0.007–0.011	0.008	0.708	−0.036–0.053
Pain Type: discogenic vs. radicular vs. mixed	−0.037	0.266	−0.101–0.028	−0.190	0.247	−0.511–0.132
MRI Findings: spinal stenosis vs. disc degeneration vs. disc protrusion/herniation vs. no MRI available	0.034	0.334	−0.034–0.102	0.198	0.255	−0.143–0.540
PRP Injection Site: L4–S1 vs. L3–L4 vs. L1–L3 vs. conservative treatment	0.074	0.167	−0.031–0.179	0.438	0.102	−0.087–0.963
Transforaminal epidural PRP injections vs. intradiscal injections vs. conservative	−0.048	0.626	−0.242–0.146	−0.237	0.631	−1.204–0.730
Baseline NRS	−0.001	0.986	−0.062–0.061	0.016	0.919	−0.323–0.291
Baseline ODI	0.002	0.819	−0.018–0.023	0.012	0.813	−0.089–0.113
Baseline GAD-7	0.008	0.702	−0.031–0.047	0.032	0.749	−0.165–0.229
Baseline PHQ-9	0.004	0.863	−0.040–0.048	0.035	0.757	−0.186–0.256

Abbreviations: PRP, platelet-rich plasma; 95% CI, 95% confidence interval; NRS, Numeric Rating Scale; ODI, Oswestry Disability Index; GAD-7, Generalized Anxiety Disorder-7; PHQ-9, Patient Health Questionnaire-9.

## Data Availability

The original contributions presented in this study are included in the article. Further inquiries can be directed to the corresponding author.

## Appendix A

**Table A1:** STROBE checklist.

STROBE Item	Recommendation	Response/Manuscript Location	Reported on Page and Line No.
1a	Title/abstract identify study design	Clearly stated as retrospective cohort study	P.1 Line 1–35
1b	Structured abstract	Provided	P.1 Line 25–45
2	Background/rationale	Section 1	P.2–3 Line 48–112
3	Objectives	End of Introduction	P.3 Line 1–35
4	Study design	Section 2	P.3 Line 115–135
5	Setting	Section 2	P.3 Line 115–135
6a	Participants eligibility criteria	Clearly defined in Methods	P.3–4 Line 137–167
6b	Selection process	Retrospective electronic medical record (EMR) review described	P.3 Line 127–132
7	Variables	Defined (NRS, ODI, PRP vs. conservative)	P.4–6 Line 168–254
8	Data sources	EMR and clinical records	P.3 Line 121–126
9	Bias	Discussed in limitations	P.16 Line 532–554
10	Study size	Based on available eligible patients	P.5 Line 248–254
11	Quantitative variables	NRS and ODI continuous outcomes	P.5 Line 225–228
12a	Statistical methods	Repeated-measures ANOVA and a linear mixed-effects model	P.6–7 Line 255–268
12b	Confounding	Acknowledged limitation	P.16 Line 532–554
12c	Missing data	Missing outcome data and early loss to follow-up were described; these patients were excluded from the final analysis.	P.4 Line 163–167
12d	Subgroups	PRP technique subgroup analysis performed	P.12 Line 378–387
12e	Sensitivity analysis	We applied two statistical approaches, a linear mixed-effects model and repeated-measures analysis of variance (ANOVA), as part of a sensitivity analysis to assess the robustness of the findings.	P.10–12 Line 349–387
13a	Participants flow	Flow diagram included	P.6 Line 248–254
13b	Loss to follow-up	Reported	P.4 Line 165–167
14	Descriptive data	Table 1	P.8 Line 309–310
15	Outcome data	Table 2 longitudinal outcomes	P.11 Line 364–365.
16	Main results	Group/time/interaction effects reported	P.9–12 Line 310–387
16b	Effect sizes/CI	95% CIs added	P.8–10 Line 309–347
17	Other analyses	Subgroup analysis reported	P.19 Line 598–599
18	Key results summary	3.4. Clinical relevance of the results	P.10 Line 337–347
19	Limitations	Clearly expanded	P.16 Line 532–554
20	Interpretation	Revised to cautious tone	P.17 Line 557–570
21	Generalisability	Discussed	P.17 Line 557–570
22	Funding/COI	Declared	P.17 Line 580–588

**Table A2:** Comparison of NRS and ODI Between Patients Receiving Transforaminal Epidural PRP Injections and Intradiscal PRP Injections.

	Transforaminal Epidural PRP Injections (*n* = 81)			Intradiscal PRP Injections (*n* = 231)			
	Mean	SD	95% CI	Mean	SD	95% CI	*p* Value
NRS (at 0 month)	7.15	0.92	6.94–7.35	7.22	0.87	7.11–7.33	0.527
NRS (at 3 month)	4.45	1.00	4.24–4.68	4.53	0.71	4.44–4.62	0.487
NRS (at 6 month)	2.88	0.97	2.66–3.09	2.85	0.73	2.75–2.94	0.785
ODI (at 0 month)	36.93	3.74	36.10–37.75	37.32	2.89	36.95–37.70	0.330
ODI (at 3 month)	23.56	5.03	22.44–24.67	23.80	3.52	23.34–24.25	0.638
ODI (at 6 month)	15.67	4.90	14.59–16.75	15.26	3.62	14.80–15.73	0.430

Abbreviations: PRP, platelet-rich plasma; SD, standard deviation; 95% CI, 95% confidence interval; NRS, Numeric Rating Scale; ODI, Oswestry Disability Index.
